# Function Analysis of the Euclidean Distance between Probability Distributions

**DOI:** 10.3390/e20010048

**Published:** 2018-01-11

**Authors:** Namyong Kim

**Affiliations:** Division of Electronic & Information Communication, Kangwon National University, Samcheok 25913, Korea; namyong@kangwon.ac.kr; Tel.: +82-01-7188-5872

**Keywords:** distribution, recursive, gradient, spreading, functions

## Abstract

Minimization of the Euclidean distance between output distribution and Dirac delta functions as a performance criterion is known to match the distribution of system output with delta functions. In the analysis of the algorithm developed based on that criterion and recursive gradient estimation, it is revealed in this paper that the minimization process of the cost function has two gradients with different functions; one that forces spreading of output samples and the other one that compels output samples to move close to symbol points. For investigation the two functions, each gradient is controlled separately through individual normalization of each gradient with their related input. From the analysis and experimental results, it is verified that one gradient is associated with the role of accelerating initial convergence speed by spreading output samples and the other gradient is related with lowering the minimum mean squared error (MSE) by pulling error samples close together.

## 1. Introduction

Adaptive signal processing is carried out by minimizing or maximizing an appropriate performance criterion for adjusting weights of algorithms designed based on that criterion [1]. The mean squared error (MSE) criterion that measures the average of the squares of the error signal is widely employed in the Gaussian noise environment. However in non-Gaussian noise like impulsive noise, the averaging process of squared error samples that may mitigate the effects of the Gaussian noise is defeated because a single large, impulse can dominate these sums. As recent signal processing methods, the information-theoretic learning (ITL) is based on the information potential concept that data samples can be treated as physical particles in an information potential field where they interact with each other by information forces [2]. The ITL method usually exploits probability distribution functions constructed by the kernel density estimation method with the Gaussian kernel.

Among the ITL criteria, Euclidian distance (ED) between two distributions has been known to be effective in signal processing fields demanding similarity measure functions [3,4,5]. For training of adaptive systems for medical diagnosis, the ED criterion has been successfully applied to distinguish biomedical datasets [6]. For finite impulse response (FIR) adaptive filter structures in impulsive noise environments, ED between the output distribution and a set of Dirac delta functions has been used as an efficient performance criterion taking advantage of the outlier-cutting effect of Gaussian kernel for output pairs and symbol-output pairs [7]. In this approach with output distribution and delta functions, minimization of the ED (MED) leads to adaptive algorithms that adjust weights so as for the output distribution to be formed into the shape of delta functions located at each symbol point, that is, output samples concentrate on symbol points. Though the blind MED algorithm shows superior performance of robustness against impulsive noise and channel distortions, a drawback of heavy computational burden lies in it. The computational complexity is due in large part to the double summation operations at each iteration time for its gradient estimation. A follow-up study [8], however, shows that the drawback can be reduced significantly by employing a recursive gradient estimation method.

The gradient in ED minimization process of the MED algorithm has two components; one for kernel function of output pairs and the other for kernel function of symbol-output pairs. The roles of these two components have not been investigated or analyzed in scientific literature. In this paper, we analyze the roles of the two components and prove the analysis through controlling each component individually by normalizing each component with component-related input power. Through simulation in multipath channel equalization under impulsive noise, their roles of managing sample pairs are verified, and it is shown that the proposed method of controlling each component through power normalization increases convergence speed and lowers steady state MSE significantly in multipath and impulsive noise environment.

## 2. MSE Criterion and Related Algorithms

Employing the tapped delay line (TDL) structure, the output *y_k_* becomes $y_{k}=W_{k}^{T}X_{k}$ at time *k* with the input vector $X_{k}={\left[x_{k},x_{k-1},\ldots ,x_{k-L+1}\right]}^{T}$ and weight $W_{k}={\left[w_{0.k},\;w_{1,k},\;\ldots ,\;w_{L-1,k}\right]}^{T}$. Given the desired signal *d_k_* chosen randomly among the *M* symbol points (*A*_1_, *A*_2_, …, *A_M_*), the system error is calculated as *e_k_* = *d_k_* − *y_k_*. In blind equalization, the constant modulus error $e_{\mathrm{CME},k}={\left|y_{k}\right|}^{2}-R_{2}$ where $R_{2}=E\left[{\left|d_{k}\right|}^{4}\right]/E\left[{\left|d_{k}\right|}^{2}\right]$ is mostly used [9].

The MSE criterion, one of the most widely used criteria, is the statistical average *E*[·] of error power $e_{k}^{2}$ in supervised equalization and of CME power ${\left({\left|y_{k}\right|}^{2}-R_{2}\right)}^{2}$ in a blind one. For practical implementation we can use the instant squared error $e_{k}^{2}$ as a cost function in supervised equalization. With the gradient $\frac{\partial e_{k}^{2}}{W}=-2e_{k}X_{k}$ and a step size *μ_LMS_*, minimization of $e_{k}^{2}$ leads to the least mean square (LMS) algorithm [1]:

(1) $$W_{k+1}=W_{k}-\mu_{\mathrm{LMS}}\frac{\partial e_{k}^{2}}{\partial W}=W_{k}+\mu_{\mathrm{LMS}}2e_{k}X_{k}$$

As an extension of the LMS algorithm, the normalized LMS (NLMS) algorithm has been introduced where the gradient is normalized as proportional to the inverse of the dot product of the input vector with itself ${\left\|X_{k}\right\|}^{2}=X_{k}^{T}X_{k}=\sum_{m=0}^{L-1}x_{k-m}^{2}$ as a result of minimizing weight perturbation ${\left\|W_{k+1}-W_{k}\right\|}^{2}$ of the LMS algorithm [1]. Then the NLMS algorithm becomes:

(2) $$W_{k+1}=W_{k}+\mu_{\mathrm{NLMS}}\frac{e_{k}X_{k}}{\sum_{m=0}^{L-1}x_{k-m}^{2}}$$

The NLMS algorithm is known to be more stable with unknown signals and effective in real time adaptive systems [10,11]. We can see under impulsive noise environments that a single large error sample induced by impulsive noise can generate large weight perturbations. The perturbation becomes zero only when the error *e_k_* is zero. So we can predict that the weight update process (1) may be unstable so that it requires a very small step size in impulsive noise environment. Also the LMS and NLMS algorithms utilizing instant error power $e_{k}^{2}$ may cause instability in an impulsive noise environment.

## 3. ED Criterion and Entropy

Unlike the MSE based on error power, probability distribution functions can be used in constructing performance criterion. As one of the criteria utilizing distributions, the ED between the distribution of transmitted symbol *f_D_*(*d*) and the equalizer output distribution *f_Y_*(*y*) is defined as (3) [3,6].

(3) $$\mathrm{ED}=\int {\left[f_{D}^{}(\alpha )-f_{Y}^{}(\alpha )\right]}^{2}d\alpha$$

Assuming that modulation schemes are known to receivers beforehand and all the *M* symbol points (*A*_1_, *A*_2_, …, *A_M_*) are equally likely, the distribution of the transmitted symbols can be expressed as:

(4) $$f_{D}(\alpha )=\frac{1}{M}[\delta (\alpha -A_{1})+\delta (\alpha -A_{2})+\ldots +\delta (\alpha -A_{m})+\ldots +\delta (\alpha -A_{M})]$$

The output distribution can be estimated based on kernel density estimation method $f_{Y}\left(y\right)=1/N\sum_{i=1}^{N}G_{\sigma }\left(y-y_{i}\right)$ with a set of available *N* output samples {*y*_1_, *y*_2_, …, *y_N_*} [6].

Then the ED can be expressed as:

(5) $$\mathrm{ED}=\frac{1}{M}+\frac{1}{N^{2}}\sum_{i=1}^{N}\sum_{j=1}^{N}G_{\sigma \sqrt{2}}(y_{j}-y_{i})-2\frac{1}{M}\frac{1}{N}\sum_{m=1}^{M}\sum_{i=1}^{N}G_{\sigma }(A_{m}-y_{i})$$

The first term 1/*M* in (5) is a constant which is not adjustable, so the ED can be reduced to the following performance criterion *C*_ED_ [7]:

(6) $$C_{\mathrm{ED}}=\frac{1}{N^{2}}\sum_{i=1}^{N}\sum_{j=1}^{N}G_{\sigma \sqrt{2}}(y_{j}-y_{i})-2\frac{1}{M}\frac{1}{N}\sum_{m=1}^{M}\sum_{i=1}^{N}G_{\sigma }(A_{m}-y_{i})$$

In ITL methods, data samples are treated as physical particles interacting with each other. If we place physical particles in the locations of *y_i_* and *y_j_*, the Gaussian kernel $G_{\sigma \sqrt{2}}\left(y_{j}-y_{i}\right)$ produces an exponentially decaying positive value as the distance between the two particles increases. This leads us consider the Gaussian kernel $G_{\sigma \sqrt{2}}\left(y_{j}-y_{i}\right)$ as a potential field-inducing interaction among particles. Then $\sum_{j=1}^{N}G_{\sigma \sqrt{2}}\left(y_{j}-y_{i}\right)$ corresponds to the sum of interactions on the *i*-th particle and $1/N^{2}\sum_{i=1}^{N}\sum_{j=1}^{N}G_{\sigma \sqrt{2}}\left(y_{j}-y_{i}\right)$ is the averaged sum of all pairs of interactions. This summed potential energy is referred to as information potential in ITL methods [2]. Therefore, the term $\frac{1}{M}\frac{1}{N}\sum_{m=1}^{M}\sum_{i=1}^{N}G_{\sigma }\left(A_{m}-y_{i}\right)$ in (6) is the information potential between symbol points and output samples, and $1/N^{2}\sum_{i=1}^{N}\sum_{j=1}^{N}G_{\sigma \sqrt{2}}\left(y_{j}-y_{i}\right)$ in (6) indicates the information potential among output samples themselves.

On the other hand, the information potential can be interpreted in the concept of entropy that can be described in terms of “energy dispersal” or the “spreading of energy” [11]. As one of the convenient entropy definitions, Reny’s entropy of order 2, *H*_Reny_(*y*) is defined in (7) as logarithm of the sum of the power of probability which is much easier to estimate [2]:

(7) $$H_{\mathrm{Reny}}(y)=-\log(\sum_{i=1}^{N}p_{i}^{2})$$

When the Reny’s entropy is used along with the kernel density estimation method $f_{Y}\left(y\right)=1/N\sum_{i=1}^{N}G_{\sigma }\left(y-y_{i}\right)$, we obtain a much simpler form of Reny’s quadratic entropy as:

(8) $$H_{\mathrm{Reny}}(y)=-\log(\frac{1}{N^{2}}\sum_{i=1}^{N}\sum_{j=1}^{N}G_{\sigma \sqrt{2}}(y_{j}-y_{i}))$$

This leads to:

(9) $$\frac{1}{N^{2}}\sum_{i=1}^{N}\sum_{j=1}^{N}G_{\sigma \sqrt{2}}(y_{j}-y_{i})=\frac{1}{2^{H_{\mathrm{Reny}}(y)}}$$

Likewise:

(10) $$\frac{1}{M}\frac{1}{N}\sum_{m=1}^{M}\sum_{i=1}^{N}G_{\sigma }(A_{m}-y_{i})=\frac{N}{M}\frac{1}{2^{H_{\mathrm{Reny}}(Am,x)}}$$

Therefore the cost function *C*_ED_ becomes:

(11) $$C_{\mathrm{ED}}=\frac{1}{2^{H_{\mathrm{Reny}}(y)}}-2\frac{N}{M}\frac{1}{2^{H_{\mathrm{Reny}}(Am,x)}}$$

Equations (9) and (11) indicate that the entropy of output samples increases as the distance (*y_j_* − *y_i_*) between the two information particles *y_j_* and *y_i_* increases. Therefore, (*y_j_* − *y_i_*) can be referred to as entropy-governing output and we can notice that (9) controls the spreading of output samples. Likewise, the term $2\frac{1}{M}\frac{1}{N}\sum_{m=1}^{M}\sum_{i=1}^{N}G_{\sigma }\left(A_{m}-y_{i}\right)$ in (6), that is, $2\frac{N}{M}\frac{1}{2^{H_{Reny}\left(A_{m},x\right)}}$ in (11) governs dispreading or recombining the sample pairs of symbol points and output samples.

## 4. Entropy-Governing Variables and Recursive Algorithms

When defining *y_j,i_* = (*y_j_* − *y_i_*) and *e_m,i_* = (*A_m_* – *y_i_*) and **X***_j.i_* = (**X*_j_*** – **X***_i_*) for convenience’s sake, *y_j,i_*, *e_m,i_* and **X***_j,i_* can be referred to as entropy-governing output, entropy-governing error and entropy-governing input, respectively. Using these entropy-governing variables and the on-line density estimation method $f_{X.k}\left(y\right)=\frac{1}{N}\sum_{i=k-N+1}^{k}G_{\sigma }\left(y-y_{i}\right)$ instead of *f_Y_*(*y*), the cost function at time *k*, *C*_ED,*k*_ can be written as:

(12) $$C_{\mathrm{ED},k}=U_{k}-V_{k}$$

where:

(13) $$U_{k}=\frac{1}{2^{H_{\mathrm{Reny}}(y)}}=\frac{1}{N^{2}}\sum_{i=k-N+1}^{k}\sum_{j=k-N+1}^{k}G_{\sigma \sqrt{2}}(y_{j,i})$$

(14) $$V_{k}=2\frac{N}{M}\frac{1}{2^{H_{Reny}(Am,x)}}=2\frac{1}{M}\frac{1}{N}\sum_{i=k-N+1}^{k}\sum_{j=k-N+1}^{k}G_{\sigma }(e_{m,}{}_{i})$$

Minimization of *C*_ED,*k*_ indicates that *U_k_* forces spreading of output samples and −*V_k_* compels output samples to move close to symbol points. Considering that initial-stage output samples which may have clustered about wrong places due to channel distortion, *U_k_* is associated with the role of getting the output samples to move out in search of each destination, that is, accelerating initial convergence speed. On the other hand, *V_k_* is related with compelling output samples near a symbol point to come close lowering the minimum MSE.

On the other hand, the double summation operations for *U_k_* and *V_k_* impose a heavy computational burden. In the work [8] it has been revealed that each component *U_k_*_+1_ and *V_k_*_+1_ of *C*_ED,*k*+1_ = *U_k_*_+1_ − *V_k_*_+1_ can be recursively calculated so that the computational complexity of (12) is significantly reduced as in the following equations (15) and (16):

(15) $$U_{k+1}^{}=U_{k}^{}+\frac{2}{N^{2}}\sum_{j=k-N+1}^{k}G_{\sigma \sqrt{2}}(y_{i,k+1})-\frac{2}{N^{2}}\sum_{j=k-N+1}^{k}\frac{1}{2\sigma \sqrt{\pi }}\exp[\frac{-{\left(y_{i,k-N+1}\right)}^{2}}{4\sigma^{2}}]\;-\frac{2}{N^{2}}\frac{1}{2\sigma \sqrt{\pi }}\exp[\frac{-{\left(y_{k+1,k-N+1}\right)}^{2}}{4\sigma^{2}}]+\frac{2}{N^{2}}\frac{1}{2\sigma \sqrt{\pi }}$$

Similarly, *V_k_*_+1_ can be divided into the terms with *y_k_*_+1_ and the terms with *y_k−N_*_+1_:

(16) $$V_{k+1}^{}=V_{k}^{}+\frac{2}{NM}\sum_{m=1}^{M}[\frac{1}{\sigma \sqrt{2\pi }}\exp[\frac{-{\left(e_{m,k+1}\right)}^{2}}{2\sigma^{2}}]-\frac{1}{\sigma \sqrt{2\pi }}\exp[\frac{-{\left(e_{m,k-N+1}\right)}^{2}}{2\sigma^{2}}]]$$

The gradients ∂Uk∂W and ∂Vk∂W are calculated recursively by using Equations (15) and (16) as:

(17) $$\begin{array}{l} \frac{\partial U_{k}^{}}{\partial W}=\frac{\partial U_{k-1}^{}}{\partial W}+\frac{1}{N^{2}\sigma^{2}}\sum_{j=k-N}^{k-1}\left(y_{k,i}\right)\cdot \frac{1}{2\sigma \sqrt{\pi }}\exp[\frac{-{\left(y_{k,i}\right)}^{2}}{4\sigma^{2}}]\cdot X_{i,k}\\ \;-\frac{1}{N^{2}\sigma^{2}}\sum_{j=k-N}^{k-1}\left(y_{k-N,i}\right)\cdot \frac{1}{2\sigma \sqrt{\pi }}\exp[\frac{-{\left(y_{k-N,i}\right)}^{2}}{4\sigma^{2}}]\cdot X_{i,k-N}\\ \;-\frac{1}{N^{2}\sigma^{2}}(y_{k-N,k})\cdot \frac{1}{2\sigma \sqrt{\pi }}\exp[\frac{-{\left(y_{k-N,k}\right)}^{2}}{4\sigma^{2}}]\cdot X_{k,k-N} \end{array}$$

Similarly, ∂Vk∂W is calculated recursively as described below:

(18) $$\begin{array}{l} \frac{\partial V_{k}^{}}{\partial W}=\frac{\partial V_{k-1}^{}}{\partial W}+\frac{2}{NM\sigma^{2}}\sum_{m=1}^{M}[(e_{m,k})\cdot \frac{1}{\sigma \sqrt{2\pi }}\exp[\frac{-{\left(e_{m,k}\right)}^{2}}{2\sigma^{2}}]\cdot X_{k}\\ \;-(e_{m,k-N})\cdot \frac{1}{\sigma \sqrt{2\pi }}\exp[\frac{-{\left(e_{m,k-N}\right)}^{2}}{2\sigma^{2}}]\cdot X_{k-N}] \end{array}$$

Since the argument $y_{k,i}\frac{1}{2\sigma \sqrt{\pi }}\exp\left[\frac{{\left(y_{k,i}\right)}^{2}}{4\sigma^{2}}\right]$ in (17) is a function of the entropy-governing output *y_k,i_*, we can define $y_{k,i}\frac{1}{2\sigma \sqrt{\pi }}\exp\left[\frac{{\left(y_{k,i}\right)}^{2}}{4\sigma^{2}}\right]$ as the modified entropy-output ${\hat{y}}_{k,i}$, which becomes a significantly mitigated value through the Gaussian kernel when the entropy-governing output *y_k,i_* is a large value.

(19) $$\overset{\wedge }{y_{k,i}}=y_{k,i}\frac{1}{2\sigma \sqrt{\pi }}\exp[\frac{-{\left(y_{k,i}\right)}^{2}}{4\sigma^{2}}]$$

Then (17) becomes

(20) $$\frac{\partial U_{k}^{}}{\partial W}=\frac{\partial U_{k-1}^{}}{\partial W}+\frac{1}{N^{2}\sigma^{2}}\sum_{j=k-N}^{k-1}\overset{\wedge }{y_{k,i}}\cdot X_{i,k}-\frac{1}{N^{2}\sigma^{2}}\sum_{j=k-N}^{k-1}\overset{\wedge }{y_{k-N,i}}\cdot X_{i,k-N}-\frac{1}{N^{2}\sigma^{2}}\overset{\wedge }{y_{k-N,k}}\cdot X_{k,k-N}$$

Similarly, we see that the argument $e_{m,k}\frac{1}{\sigma \sqrt{2\pi }}\exp\left[\frac{-{\left(e_{m,k}\right)}^{2}}{2\sigma^{2}}\right]$ in (18) is a function of entropy-governing error *e_m,k_*, so that we have the modified entropy-error ${\hat{e}}_{m,k}$ as:

(21) $$\overset{\wedge }{e_{m,k}}=e_{m,k}\cdot \frac{1}{\sigma \sqrt{2\pi }}\exp[\frac{-{\left(e_{m,k}\right)}^{2}}{2\sigma^{2}}]$$

The modified entropy-error ${\hat{e}}_{m,k}$ also becomes a significantly reduced value through the Gaussian kernel when the entropy-governing error *e_m,k_* is large. Then (18) becomes:

(22) $$\frac{\partial V_{k}^{}}{\partial W}=\frac{\partial V_{k-1}^{}}{\partial W}+\frac{2}{NM\sigma^{2}}\sum_{m=1}^{M}[\overset{\wedge }{e_{m,k}}\cdot X_{k}-\overset{\wedge }{e_{m,k-N}}\cdot X_{k-N}]$$

Through minimization of *C*_ED,*k*_ = *U_k_* − *V_k_* with the gradients ∂Uk∂W and ∂Vk∂W obtained by (20) and (22), the following recursive MED (RMED) algorithm can be derived [7]:

(23) $$W_{k+1}=W_{k}-\mu_{\mathrm{RMED}}\frac{\partial (U_{k}-V_{k})}{\partial W}=W_{k}-\mu_{\mathrm{RMED}}(\frac{\partial U_{k}}{\partial W}-\frac{\partial V_{k}}{\partial W})$$

Comparing the gradients of RMED to the gradient ∂ek2∂W=−2ekXk of the LMS algorithm in (1) which is composed of error and input, we may find that the gradients ∂Uk∂W and ∂Vk∂W in (20) and (22) have similar terms ${\hat{y}}_{k,i}\cdot X_{i,k}$ (modified entropy-output multiplied by entropy-input) and ${\hat{e}}_{m,k}\cdot X_{k}$ (modified entropy-error multiplied by input), respectively. Considering that impulsive noise may induce large entropy-governing output *y_k,i_* or entropy-governing error *e_m,k_*, modified entropy-output ${\hat{y}}_{k,i}$ and modified entropy-error ${\hat{e}}_{m,k}$ which are significantly mitigated by the Gaussian kernel can be viewed as playing a crucial role in obtaining stable gradients under strong impulsive noise. Therefore we can anticipate that the RMED algorithm (23) can have a low weight perturbation in impulsive noise environments.

## 5. Input Power Estimation for Normalized Gradient

For the purpose of minimizing the weight perturbation ${\left\|W_{k+1}-W_{k}\right\|}^{2}$ of the LMS algorithm in (1), the *NLMS* algorithm has been introduced where the gradient is normalized by the averaged power of the current input samples ${\left\|X_{k}\right\|}^{2}=X_{k}^{T}X_{k}=\sum_{m=0}^{L-1}x_{k-m}^{2}$ [1].

(24) $$W_{k+1}=W_{k}+\mu_{\mathrm{NLMS}}\frac{e_{k}X_{k}}{{\left\|X_{k}\right\|}^{2}}$$

Applying this approach to RMED we propose in this section to normalize the gradients in some ways. Since the role of *U_k_* (spreading output samples) is different from that of *V_k_* (moving output samples close to symbol points), the gradients of (23) can be normalized separately as:

(25) $$W_{k+1}=W_{k}-\mu_{\mathrm{RMED}}\frac{\partial U_{k}}{\partial W}\frac{1}{P_{U}(k)}+\mu_{\mathrm{RMED}}\frac{\partial V_{k}}{\partial W}\frac{1}{P_{V}(k)}$$

where *P_U_*(*k*) is the average power of **X***_i,k_* and *P_V_*(*k*) is the average power of **X***_k_* as:

(26) $$P_{U}(k)=\frac{1}{N}\sum_{i=k-N+1}^{k}\sum_{j=k-N+1}^{k}{\left|x_{i,j}^{}\right|}^{2}$$

(27) $$P_{V}(k)=\frac{1}{N}\sum_{i=k-N+1}^{k}{\left|x_{i}^{}\right|}^{2}$$

Since defeating the impulsive noise contained in the input by way of the average operation $\frac{1}{N}\sum_{i=k-N+1}^{k}$ is considered to be ineffective, the denominators of (26) and (27) are likely to be fluctuating under impulsive noise. This may cause the algorithm to be sensitive to impulsive noise. Also the summation operators make the algorithm demand computationally burdensome. To avoid these drawbacks, we can track the average power *P_U_*(*k*) and *P_V_*(*k*) recursively with the balance parameter *β* (0 < *β* <1) as:

(28) $$P_{U}(k)=\beta P_{U}(k-1)+(1-\beta )\sum_{j=k-N+1}^{k}{\left|x_{i,j}^{}\right|}^{2}$$

(29) $$P_{V}(k)=\beta P_{V}(k-1)+(1-\beta ){\left|x_{k}^{}\right|}^{2}$$

With the recursive power estimation (28) and (29), we may summarize the proposed algorithm in a more formal one as in the Table 1. In the following section, we will investigate the new RMED algorithm (25) with separate normalization by *P_U_*(*k*) in (28) and *P_V_*(*k*) in (29) in the aspect of convergence speed and steady state MSE.

## 6. Results and Discussion

**Figure 1:**
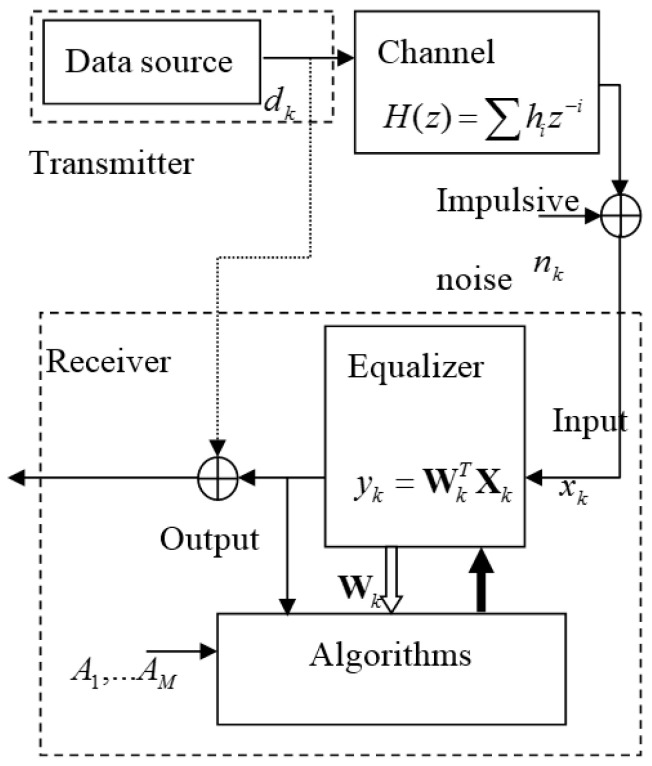
Base-band communication system for simulation.

A base-band communication system with multipath fading channel and impulsive noise used in the experiment is depicted in Figure 1. The symbol set in the transmitter is composed of equally probable four symbols (−3, −1, 1, 3). The transmitted symbol is to be distorted by the multipath channel *H*(*z*) = 0.26 + 0.93*z*^−1^ + 0.26*z*^−2^ [12]. The channel output is added by impulsive noise *n_k_*. The distribution function of *n_k_*, *f*(*n_k_*) is expressed in Table 2 where $\sigma_{IN}^{2}$ is the variance of impulses which are generated according to Poisson process (occurrence rate *ε*) and $\sigma_{GN}^{2}$ is that of the background Gaussian noise [13]. The simulation setup and parameter values are described in the Figure 1 and the Table 2.

**Figure 2:**
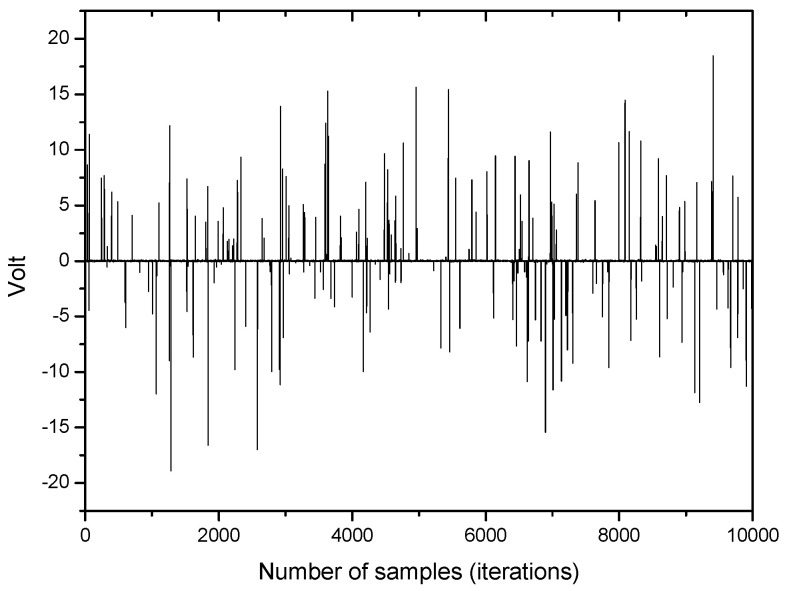
An example of impulsive noise.

An example of impulsive noise being used in this simulation is depicted in Figure 2.

It has in Section 4 been analyzed that *U_k_* is associated with the role of spreading output samples which are clustered to wrong positions due to distorted channel characteristics and *V_k_* is related with moving output samples close to symbol points. This process can be explained through initial-stage investigation of what happens in the error distribution and observing how the distribution of output samples changes in the experimental environment.

**Figure 3:**
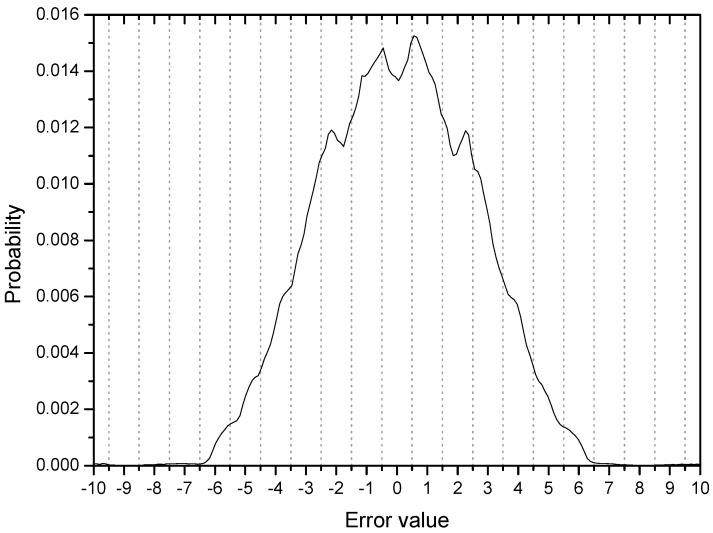
The error distribution at time *k* = 200 with 200 error samples.

**Figure 4:**
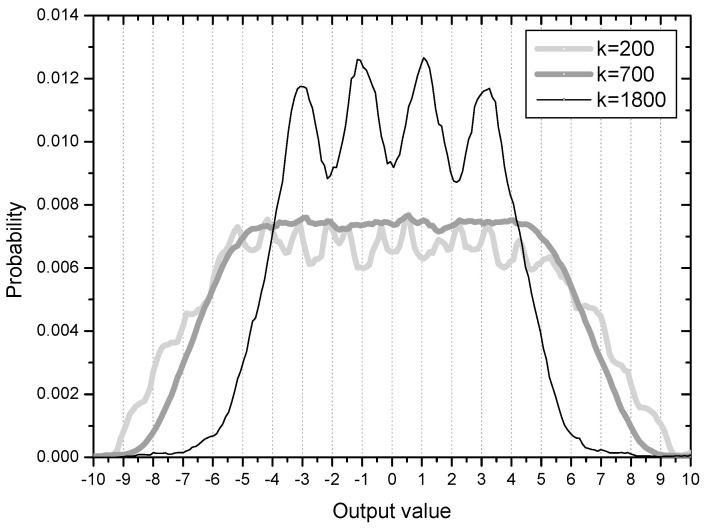
Output distributions in an initial stage.

Figure 3 shows the error distribution in the initial stage with 200 error samples and ensemble average of 500 runs. Considering the four symbol points are (−3, −1, 1, 3), error values greater than 1.0 are associated with output samples which can be decided as wrong symbols. The cumulative probability of initial output samples placed in the wrong regions in this respect is calculated to be 0.35 from the Figure 3 (35% output samples are not in place). The peaks or ridges in the error distribution are about 6 on each side. This observation may indicate that output samples are clustered or grouped in some regions (two groups are within the correct range but 4 groups are in the incorrect positions on each side of the distribution). This result coincides clearly with the initial output distribution in Figure 4. The output distribution showing about 12 peaks indicates that the initial output samples are clustered into 12 groups mostly located out of place, that is, not around −3, −1, 1, 3.

On the 35% output samples clustered in the wrong symbol regions, the spreading force has a positive effect in order for them in blind search to move out for finding their correct symbol positions. This process is observed in the graph of *k* = 700 in Figure 4. The output distribution at time *k* = 700 has an evenly spread shape, indicating that the clustered output samples have moved out and mingled with one another. At the sample time *k* = 1800 the output samples start to position at their correct symbol areas. From this phase, the force moving output samples close to the symbol points is in effect on lowering steady state MSE.

These results imply that *U_k_* is related with convergence speed and *V_k_* with steady state MSE. To verify this analysis we experiment the proposed algorithm in the following three modes with respect to convergence speed and steady state MSE (we assume that steady state MSE is close to minimum MSE):

(30) $$\mathrm{Mode}\;1\;W_{k+1}=W_{k}-\mu_{\mathrm{RMED}}\frac{\partial U_{k}}{\partial W}\frac{1}{P_{U}(k)}+\mu_{\mathrm{RMED}}\frac{\partial V_{k}}{\partial W}$$

(31) $$\mathrm{Mode}\;2\;W_{k+1}=W_{k}-\mu_{\mathrm{RMED}}\frac{\partial U_{k}}{\partial W}+\mu_{\mathrm{RMED}}\frac{\partial V_{k}}{\partial W}\frac{1}{P_{V}(k)}$$

(32) $$\mathrm{Mode}\;3\;W_{k+1}=W_{k}-\mu_{\mathrm{RMED}}\frac{\partial U_{k}}{\partial W}\frac{1}{P_{U}(k)}+\mu_{\mathrm{RMED}}\frac{\partial V_{k}}{\partial W}\frac{1}{P_{V}(k)}$$

Mode 1 of RMED-SN algorithm in (30) is for observing changes in initial convergence speed by normalizing only ∂Uk∂W by the average power *P_U_*(*k*) of entropy-input **X***_i,k_* compared to the not-normalized RMED. Mode 2 is to observe whether the normalization of ∂Vk∂W by *P_V_*(*k*) of input **X***_k_* without managing *U_k_* lowers the steady state MSE of RMED. Finally we see if Mode 3 employing normalization of ∂Uk∂W and ∂Vk∂W simultaneously yields both of the two performance enhancements; faster convergence and lowered steady state MSE.

**Figure 5:**
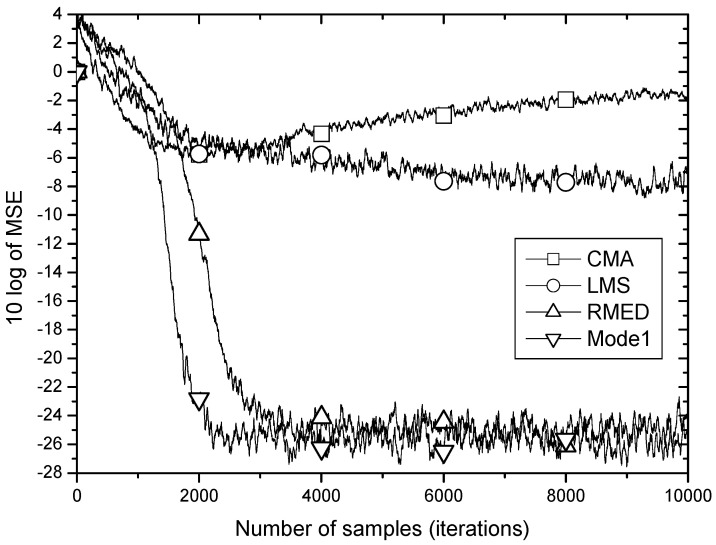
MSE convergence performance for *U_k_* normalization.

Figure 5 shows the MSE learning performance for CMA, LMS, RMED and Mode 1 of the proposed algorithm. As discussed in Section 2, the learning curves of the MSE-based algorithms, CMA and LMS do not fall down below −6 dB being defeated by the impulsive noise. On the other hand, the RMED and proposed algorithm show a rapid and stable convergence. The difference of convergence speed between RMED and Mode 1 is clearly observed. While the RMED converges in about 4000 samples, the Mode 1 does in about 2000 samples. Therefore, Mode 1 shows faster convergence than the RMED algorithm by 2 times verifying the analysis of the role of $U_{k}$ since only ∂Uk∂W is normalized but ∂Vk∂W is not, and we see little difference (about 1 dB) in the steady state MSE.

**Figure 6:**
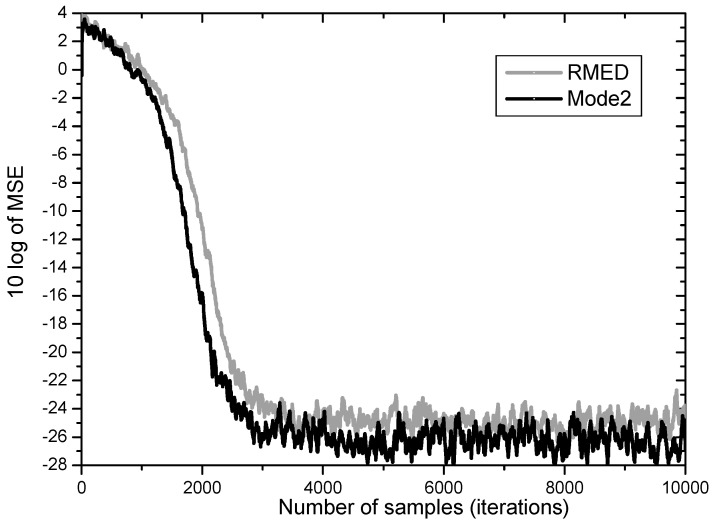
MSE convergence performance for normalization of *V_k_*.

In Figure 6 RMED and Mode 2 are compared. Both algorithms have similar convergence speed with difference of only 500 samples. But after convergence the Mode 2 yields much lower steady state MSE than the original RMED by over 2 dB. These findings indicate that the role of *V_k_* is definitely related with lowering minimum MSE. This is in accordance with the analysis that *U_k_* plays the role of pulling error samples close together.

**Figure 7:**
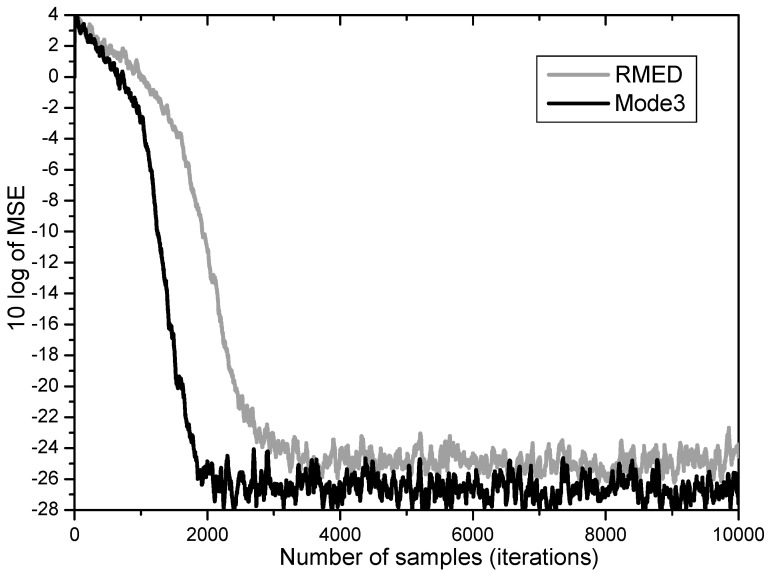
MSE convergence performance for normalization of both *U_k_* and *V_k_.*

Furthermore, Mode 3 employing normalization of ∂Uk∂W and ∂Vk∂W simultaneously proves to yield the two merits of performance enhancement revealing increased speed and lowered steady state MSE as depicted in Figure 7. While the RMED converges in about 4000 samples and leaves its steady state MSE at about 25 dB, the Mode 3 converges in about 2000 samples and has about 27 dB of steady state MSE. By employing Mode 3, we obtained faster convergence by about 2 times and lower steady state MSE by over 2 dB.

In Mode 3, it is still not clear whether the normalization to *U_k_* for speeding up the initial convergence may have a negative influence in later iterations, so we try to reduce the *U_k_* normalization gradually after convergence (*k* ≥ 3000) by using $P_{U}^{\circ }\left(k\right)$ in place of *P_U_*(*k*) as:

(33) $$P_{U}^{\circ }(k)=P_{U}(k)\cdot c^{k-3000}+(1-c^{k-3000})$$

where *k* ≥ 3000 and a constant *c* is 0 ≤ *c* ≤ 1.

**Figure 8:**
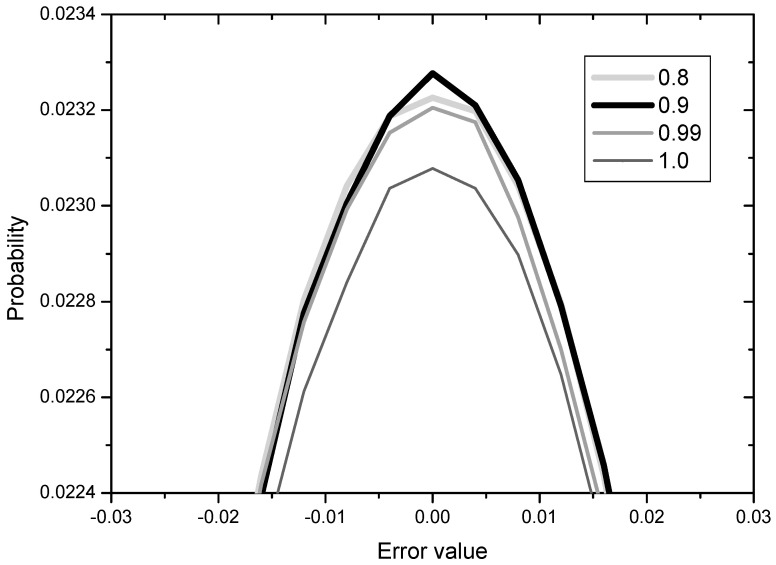
Error distribution with respect to the values of *c* for normalization of *U_k_*.

The results for *c* = 0.8, 0.9, 0.99, 1.0 are shown in Figure 8 in terms of error distribution since the learning curves for the various constant values are not clearly distinguishable.

The value of *c* in (33) may be related with the degree of gradual reduction in the normalization to *U_k_*, that is, *c* = 1 indicates no reduction (Mode 3 as it is) and *c* = 0.8 means comparatively rapid reduction. From the Figure 8, we observe that the error performance becomes better and then worse as the degree of reduction decreases from 0.8 to 1.0. This implies that the gradual reduction of the normalization to *U_k_* is effective but not much. We may conclude that the normalization to *U_k_* for speeding up the initial convergence has a slight negative influence in later iterations and this can be overcome by employing the gradual reduction of the *U_k_* normalization.

## 7. Conclusions

Minimization of the Euclidean distance between output distribution and Dirac delta function as a performance criterion is known to force the distribution of system output to come to a set of delta functions located at each symbol point. In the analysis of the algorithm RMED developed based on that criterion and recursive gradient estimation, it has been revealed in this paper that the minimization process of the cost function uses its two gradients with different functions; one for *U_k_* that forces spreading of output samples and the other one for *V_k_* that compels output samples to move close to symbol points. In order to verify the roles of *U_k_* and *V_k_* explained in the analysis by controlling *U_k_* and *V_k_* separately, we proposed to normalize ∂Uk∂W with the averaged power of entropy-governing input and to normalize ∂Vk∂W with that of input. From the results through simulation for the separate normalization of the gradients of RMED in multipath channel equalization under impulsive noise, faster convergence by about two times through normalization of ∂Uk∂W and lower steady state MSE by over 2 dB by normalization of ∂Vk∂W have been observed. From the analysis and experimental results, we can conclude that *U_k_* is associated with the role of accelerating initial convergence speed by spreading output samples which may have clustered around wrong places in the initial-stage due to channel distortions, and *V_k_* is related with lowering the minimum MSE by pulling error samples close together through the minimization of *C*_ED,*k*_. Also it can be concluded that through applying normalization to the two factors ∂Uk∂W and ∂Vk∂W separately with each related input power, significant performance enhancement can be achieved.

## Figures and Tables

**Table 1:** A summary of the proposed algorithm.

Process	Equations
Initialization	$\frac{\partial U_{0}^{}}{\partial W}=0$, $\frac{\partial V_{0}^{}}{\partial W}=0$, $P_{U}(0)=1$, $P_{V}(0)=1$, $W_{0}={\left[0,\ldots ,0,w_{L/2,0}=1,0,\ldots ,0\right]}^{T}$
Update of gradient function $\frac{\partial U_{k}^{}}{\partial W}$	$\frac{\partial U_{k}^{}}{\partial W}=\frac{\partial U_{k-1}^{}}{\partial W}+\frac{1}{N^{2}\sigma^{2}}\sum_{j=k-N}^{k-1}\overset{\wedge }{y_{k,i}}\cdot X_{i,k}-\frac{1}{N^{2}\sigma^{2}}\sum_{j=k-N}^{k-1}\overset{\wedge }{y_{k-N,i}}\cdot X_{i,k-N}-\frac{1}{N^{2}\sigma^{2}}\overset{\wedge }{y_{k-N,k}}\cdot X_{k,k-N}$
Update of gradient function $\frac{\partial V_{k}^{}}{\partial W}$	$\frac{\partial V_{k}^{}}{\partial W}=\frac{\partial V_{k-1}^{}}{\partial W}+\frac{2}{NM\sigma^{2}}\sum_{m=1}^{M}[\overset{\wedge }{e_{m,k}}\cdot X_{k}-\overset{\wedge }{e_{m,k-N}}\cdot X_{k-N}]$
Update of $P_{U}(k)$	$P_{U}(k)=\beta P_{U}(k-1)+(1-\beta )\sum_{j=k-N+1}^{k}{\left|x_{i,j}^{}\right|}^{2}$
Update of $P_{V}(k)$	$P_{V}(k)=\beta P_{V}(k-1)+(1-\beta ){\left|x_{k}^{}\right|}^{2}$
Update of $W_{k}$	$W_{k+1}=W_{k}-\mu_{\mathrm{RMED}}\frac{\partial U_{k}^{}}{\partial W}\frac{1}{P_{U}(k)}+\mu_{\mathrm{RMED}}\frac{\partial V_{k}^{}}{\partial W}\frac{1}{P_{V}(k)}$

**Table 2:** Simulation setup and parameter values.

Features	Parameters
The symbol points in the transmitter	$\left(A_{1},\;A_{2},\;A_{3},\;A_{4})=(-3,\;-1,\;+1,\;+3\right)$
The channel transfer function H(z)	$H(z)=0.26+0.93z^{-1}+0.26z^{-2}$
The noise distribution function $f(n_{k})$	$f(n_{k})=\frac{1-\varepsilon }{\sigma_{\mathrm{GN}}^{}\sqrt{2\pi }}\exp[\frac{-n_{k}{}^{2}}{2\sigma_{\mathrm{GN}}^{2}}]+\frac{\varepsilon }{\sqrt{2\pi (\sigma_{\mathrm{GN}}^{2}+\sigma_{\mathrm{IN}}^{2})}}\exp[\frac{-n_{k}{}^{2}}{2(\sigma_{\mathrm{GN}}^{2}+\sigma_{\mathrm{IN}}^{2})}]$, ε=0.03, $\sigma_{\mathrm{GN}}^{2}=0.001$, $\sigma_{\mathrm{GN}}^{2}+\sigma_{\mathrm{IN}}^{2}=50.001$
NNumber of weights	11
4 Step size	$\mu_{\mathrm{CMA}}=0.000001$, $\mu_{\mathrm{LMS}}=0.0002$, $\mu_{\mathrm{RMED}}=0.005$
Sample size N	6
Kernel size σ	0.6
